# The impact of indicated prevention and early intervention on co-morbid eating disorder and depressive symptoms: a systematic review

**DOI:** 10.1186/s40337-014-0030-2

**Published:** 2014-11-13

**Authors:** Rachel F Rodgers, Susan J Paxton

**Affiliations:** Department of Counseling and Applied Educational Psychology, Northeastern University, Boston, USA; Laboratoire de Stress Traumatique, Universite Paul Sabatier, Toulouse, France; La Trobe University, Melbourne, Australia

**Keywords:** Eating disorders, Depression, Prevention, Early intervention, Systematic review

## Abstract

**Background:**

Depressive and eating disorder symptoms are highly comorbid. To date, however, little is known regarding the efficacy of existing programs in decreasing concurrent eating disorder and depressive symptoms.

**Methods:**

We conducted a systematic review of selective and indicated controlled prevention and early intervention programs that assessed both eating disorder and depressive symptoms.

**Results:**

We identified a total of 26 studies. The large majority of identified interventions (92%) were successful in decreasing eating disorder symptoms. However fewer than half (42%) were successful in decreasing both eating disorder and depressive symptoms. Intervention and participant characteristics did not predict success in decreasing depressive symptoms.

**Conclusions:**

Indicated prevention and early intervention programs targeting eating disorder symptoms are limited in their success in decreasing concurrent depressive symptoms. Further efforts to develop more efficient interventions that are successful in decreasing both eating disorder and depressive symptoms are warranted.

## Review

Eating disorders and depressive disorders have revealed strong associations, and their relationships contribute to the complexity, burden, and treatment resistance of these disorders [1]. In view of these factors, understanding the effects of prevention and early intervention efforts on both eating disorder and depressive symptoms is an important concern, and would help inform intervention. However, to date, little attention has been paid to the success of interventions in decreasing both of these concerns. The aim of the present study was therefore to conduct a systematic review of indicated prevention and early intervention programs that assessed both eating disorder and depressive symptoms in order to clarify the efficacy of these programs in decreasing both types of symptomatology.

The lifetime comorbidity between eating disorders and major depressive disorders is high with estimates of 40% for Anorexia Nervosa and 50% for Bulimia Nervosa [2]. Importantly, in relation to eating disorders, comorbid depressive symptoms have been found to negatively affect the successfulness of early intervention efforts. Depressive symptoms have been found to predict attrition in self-help programs for binge eating disorder [3], and to predict drop-out from Internet interventions that have become more numerous in recent years [4]. Similarly, depressive symptoms have been found to predict poorer prognosis in self-help programs for eating disorders [3].

Several types of relationships have been hypothesized and described between eating disorder and depressive symptoms, namely that 1) eating disorder and depressive symptoms may develop simultaneously; 2) eating disorder symptoms may be a risk factor for depressive symptoms; 3) depressive symptoms may be a risk factor for eating disorder symptoms. While all of these models have received empirical support [5-7], compelling evidence from twin studies has revealed the importance of shared genetic factors in the development of comorbid eating disorder and depressive symptoms [8,9]. In view of this, some authors have argued that the evidence to date provides greatest support for a comorbidity model in which eating disorders and depression stem from both common and specific etiological factors [10].

A number of theoretical models have proposed frameworks that account for these shared and specific risk factors including biological, cognitive, emotional-regulation, and feminist models. Biological models emphasize the importance of shared genetically transmitted biological factors, as described above, and in particular the importance of dysregulation of serotonergic pathways that have an impact on both mood and eating behaviors [11]. Cognitive models highlight the role of common cognitive distortions that play a role in the development of both depressive and eating disorder symptoms through a heightened focus on negative information [12-14]. Emotion-regulation models have revealed how maladaptive strategies for regulating emotions may contribute to both depressive and eating disorders symptoms [15]. Finally, feminist models of the comorbidity between depressive and eating disorder symptoms have highlighted the gendered aspects of these concerns and viewed both as products of the restricted roles accessible to women in Western society and the objectifying male gaze [16-18].

Despite the high rates of comorbidity and the number of theoretical frameworks accounting for this co-occurrence, to date little is known regarding the common course of eating disorder and depressive symptoms within interventions for high-risk individuals or early intervention programs. Previous work exploring the effect of prevention interventions for eating disorders on negative affect (which includes depressive symptoms but also more serious forms of negative affect) revealed that programs targeting individuals at high risk of eating disorders were more successful in decreasing negative affect [19]. Furthermore, programs including participants over the age of 15, in interactive formats, and including psychoeducational and cognitive dissonance content, produced the greatest decreases in negative affect [19]. While these findings are useful, they may not be transposable to depressive symptoms in interventions for symptomatic individuals as negative affect has not been found to affect prevention intervention attrition [20,21]. Increasing our understanding of intervention effects on comorbid eating disorder and depressive symptoms is crucial as it has important clinical implications. In addition, this knowledge would convey important information regarding treatment efficiency and costs. Finally, strong theoretical bases exist for hypothesizing common underlying mechanisms accounting for eating disorder and depressive symptoms. However, a finding that interventions targeting these mechanisms (e.g., cognitive and emotional factors) were not successful in decreasing both types of symptomatology would point to a disconnect between theory and practice, and suggest a need for the theoretical accounts of the eating disorder/depression comorbidity to be revisited.

The aim of the present study was, therefore, to conduct a systematic review of interventions for high risk (indicated prevention) and symptomatic individuals (early intervention) that included both eating disorder and depression outcomes. Furthermore, consistent with previous findings [19], we expected that interventions that targeted individuals with higher initial levels of eating disorders and depressive symptoms would be most successful in decreasing both of these concerns. In addition, we hypothesized that interventions including a greater number of sessions, among younger participants (which might represent a shorter duration of symptoms), with content targeting negative thoughts and feelings would be most successful. Finally, as cognitive-behavioural therapy is widely recognized as one of the first line recommended treatments for depressive symptoms particularly among young people [22], we hypothesized that interventions implementing CBT strategies would be most successful in decreasing depressive symptoms.

## Methods

**Figure 1:**
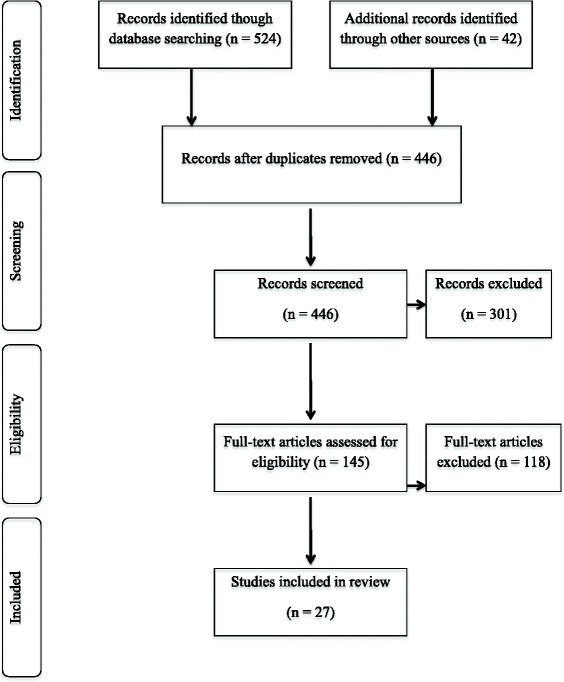
**Systematic literature review flow diagram.** Flow of information through the different phases of the systematic review.

This review was conducted in accordance to the PRISMA guidelines for systematic reviews [23]. The flow of information through different phases of the systematic review is shown in Figure 1.

### Data sources

The databases PsychInfo, ScienceDirect and Scopus were searched for studies and dissertations published between January 1984 and September 2013. Two searches were conducted in each database, first a title search, second a keyword search. The searches included combinations of words describing eating concerns (eating AND disorder or disordered or disorders or pathology or disturbance or disturbed), body image concerns (body image or dissatisfaction or shape or appearance), depressive symptoms (depression or depressive or negative affect) and controlled interventions (prevention or intervention or evaluation or program or self-help AND control or controlled). In addition, studies included in previous reviews of the efficacy of interventions aiming to reduce eating concerns as well as the reference sections of relevant articles were scanned for additional potentially eligible studies.

### Study selection

The resulting list of studies was inspected by one author to identify those meeting the following criteria:

1. Peer-reviewed published work.
2. Controlled intervention design, compared to an active or minimally active condition such as treatment as usual, or psychoeducation (randomised or not).
3. Indicated interventions defined as interventions aimed at individuals who were identified as having minimal detectable signs or symptoms of an eating but did not meet diagnostic criteria [24]. In our review we included interventions that included participants who had been screened for symptoms as well as interventions allowing participants to self-select as having high levels of concern. In addition, we included self-help and guided self-help interventions as representing the lowest intensity treatment in a stepped care model [25].
4. The concurrent assessment of both disordered eating and depressive symptoms at baseline and post-intervention, using psychometrically established measures for assessing both eating concerns and depressive symptoms.

### Data extraction

Data from the reviewed studies were extracted by one author (RR). The data extraction sheet listed the following categories designed to describe and compare the studies mean age, gender, country, inclusion criteria for the study, type of intervention, mode of delivery, depression and eating disorder symptom measure, effects of intervention at post-test and follow-up, baseline depression level, number of intervention sessions, and presence of content targeting negative thoughts and emotions.

### Data analyses

Depression scores were transformed into percentile ranks using norms from the general population [26,27]. Chi-square tests were conducted to explore the effect of initial ED levels operationalized by recruitment criteria (self-selected versus screened), CBT strategies, content targeting negative thoughts and feelings, and face-to-face delivery on successfully decreasing depressive symptoms, and successfully decreasing both depressive and eating disorder symptoms. Independent t-tests were conducted to test the effect of mean age, number of sessions, and initial levels of depressive symptoms (percentile ranks) on successfully decreasing depressive symptoms, and successfully decreasing both depressive and eating disorder symptoms. All analyses were conducted using SPSS 22.

## Results

Our review identified 26 studies corresponding to our inclusion criteria (Table 1). Of the 26, 13 studies (50%) were conducted in the U.S, 7 in Europe (27%), 4 (15%) in Australia, and 2 in Canada (8%). The age of participants ranged from 14.4 to 46.5 years old. Nineteen of the studies (73%) used the Beck Depression Inventory [28] as a measure of depressive symptoms, 3 (11%) used the Center for Epidemiology Depression Scale [29], 1 (4%) used the Montgomery-Asberg Depression Rating Scale, 1 (4%) used the Schedule for Affective Disorders and Schizophrenia for School-Age Children [30], 1 (4.33%) used the Kessler Distress Scale [31] and 1 (4%) used the Hospital Anxiety and Depression Scale [32].

**Table 1:** **Characteristics of studies included in the systematic review**

**Study**	**N intervention; N control; Gender**	**Mean age**	**Country**	**Inclusion criteria**	**Intervention; number of sessions**	**Depression assessment instrument**	***p*** **-post**	***p*** **– follow up**	**Eating disorder symptoms**	***p*** **-post**	***p*** **– follow up**
Alloway [33]	N intervention =8; N control = 6; F	32	Canada	Diabetes	Psycho-education; 6 sessions	Beck depression inventory	ns	ns		ns	ns
				EDI ≥40					EDI		
				EAT ≥17					EDI		
Banasiak [34]	N intervention =54; N control =55; F	45	Australia	meeting full or modified criteria for BN	Guided self-help vs TAU; 8 sessions	Beck depression inventory	*	N/A	EDE-Q	*	N/A
									EDI	*	N/A
Bearman [35]	N intervention =38; N control =35; F	18.9	USA	Self-selected undergrads	CBT vs control; 4 sessions	Beck depression inventory	*	ns	BPS	*	*
									DRES	*	*
									EDE-Q	*	ns
Carrard [36]	N intervention =37; N control =37; F	36	Switzerland	1 OBE a week for the past 3 months, meeting BED	Internet CBT vs control; 11 sessions	Beck depression inventory	ns	N/A	EDE-Q	*	N/A
									EDI	*	N/A
									TFEQ	*	N/A
Carrard [37]	N intervention =22; N control =20; F	42	Switzerland	BED patients 30 < BMI < 50	Internet CBT vs control; 11 sessions	Beck depression inventory	*	ns	EDE-Q	ns	ns
									TFEQ	ns	ns
Carter [38]	N intervention =28/28; N control =29; F	27	USA	BN	CBT self-help vs nonspecific self-help vs control; 8 sessions	Beck depression inventory	ns	N/A	OBE	*	N/A
									EDE-Q	ns	N/A
Fichter [39]	N intervention =68; N control =61; F	25	Germany	AN-B/P patients	Self-help vs waitlist; 6 sessions	Beck depression inventory	ns	N/A	EDI	ns	N/A
									SIAB-EX	*	N/A
Grilo [40]	N intervention =38/37; N control =15; M & F	46.5	USA	BED	CBT vs behavioural weight-loss vs control; 12 sessions	Beck depression inventory	ns	N/A	EDE-Q	*	N/A
									TFEQ	*	N/A
Grilo [41]	N intervention =24; N control =24; M & F	45.8	USA	Obese with BED	Self-help CBT vs TAU; 8 sessions	Beck depression inventory	ns	N/A	EDE-Q	*	N/A
Heinicke [42]	N intervention =36; N control =37; F	14.4	Australia	Self-selected for body image or eating concerns	CBT vs WT; 6 sessions	Beck depression inventory	*	*	BSQ	*	*
									DEBQ	*	*
									EWLB	*	*
									EDI	*	*
									SATAQ	*	*
Jacobi [43]	N intervention =51; N control =52; F	22.3	Germany	> 42 WCS	Internet CBT vs control; 8 sessions	Beck depression inventory	d = .14	d = .15	EDE-Q	d = .23-.35	d = .41-.62
									EDI	d = .08-.32	d = .35-.54
Ljotsson [44]	N intervention =35; N control =36; M & F	34	Sweden	MADRS score <30 BED or BN full or sub-threshold	Self-help CBT vs control; 12 sessions	Montgome-ry asberg depression rating scale	*	*	EDE-Q	*	*
									EDI	*	*
									BSQ	*	*
McLean [45]	N intervention =32; N control =3629 F	43.92	Australia	BSQ ≥90 or EDE-Q WSC ≥3.5	CBT vs control; 8 sessions	KDS	ns	*	BSQ	*	*
									EDEQ	*	*
									BIAQ	*	*
									SATAQ	*	*
									DEBQ	*	*
Mitchell [46]	N intervention =30/33; N control =30; F		USA	Self-selected	CD vs yoga vs control; 6 sessions	CES-D	ns	N/A	EDDS	* (CD)	N/A
									BES	ns	N/A
									EDI	* (CD)	N/A
									IBSS	ns	N/A
									TFEQ	ns	N/A
									BSQ	ns	N/A
O’Brien [47]	N intervention =13; N control =11; F	22.2	Canada	EDI > 10, skipping 2 meals a week	Control vs psycho-education; 8 sessions	Beck depression inventory	ns	ns	BULIT	*	*
									BSQ	*	ns
									EAT	*	*
									FFS	*	ns
Paxton [48]	N intervention =42/37; N control =37; F	25	Australia	BSQ >100, or BSQ =90-99 AND BULIT-R >104	CBT vs internet CBT vs waitlist; 8 sessions	Beck depression inventory	*	N/A	BSQ	*	N/A
									BULIT	*	N/A
									DEBQ	*	N/A
									BIAQ	*	N/A
									SATAQ	*	N/A
Robinson [49]	N intervention =36/34; N control =27; M & F	28	UK	BN, BED or EDNOS	eCBT vs writing vs waitlist; 24 sessions	Beck depression inventory	ns	N/A	QEDD	*	N/A
									BITE	ns	N/A
Sánchez [50]	N intervention =38; N control =38; F	23.9	UK	BN or EDNOS	iCBT vs waitlist; 8 sessions	Hospital anxiety and depression scale	N/A	*	EDE-Q	N/A	*
Stice [51]	N intervention =203; N control =205; F	21.6	USA	Self-selected and phone screened	CD vs education brochure; 4 sessions	Beck depression inventory	*	*	EDDI	*	*
									DRES	*	*
									BPS	*	*
Stice [52]	N intervention =25; N control =70; F	21.3	USA	Self-selected into eating disorders seminar	Psycho-education vs control; 28 sessions	Beck depression inventory	ns	ns	IBSS	*	*
									BPS	*	*
									DRES	*	*
									EDDS	*	*
Stice [53]	N intervention =139; N control =167; F	15.7	USA	Self-selected	CD vs control; 4 sessions	CES-D	ns	ns	IBSS	*	ns
									BPS	*	*
									DRES	*	*
									EDDI	*	*
Stice [54]	N intervention =39/19; N control =29/20; F	21.6	USA	Self-selected	CD vs ECD vs psycho-education; 4 sessions	Beck depression inventory	*	N/A	IBSS	*	N/A
									BPS	*	N/A
									DRES	*	N/A
									EDDI		N/A
Stice [55]	N intervention =198; N control =200; F	18.4	USA	Self-selected	HW vs psycho-education; 4 sessions	K-SADS	ns	ns	BDS	*	ns
									DRES	*	*
									EDDI	*	ns
Stice [56]	N intervention =55/44; N control =75; F	20.9	USA	Self-selected	CD vs Peer CD vs educational brochure; 4 sessions	Beck depression inventory	*		IBSS	*	*
									BPS	*	*
									DRES	*	*
									EDDI	*	*
Striegel [57]	N intervention =59; N control =64; M & F	37.2	USA	binge eating on average at least twice a week but missed more than one criterion for BN or BED	Guided self-help vs TAU; 8 sessions	Beck depression inventory	*	*	EDE-Q	*	*
Taylor [58]	N intervention =206; N control =215; F	20.8	USA	WCS >50	ECBT vs waitlist; 8 sessions	CES-D	ns	ns (.07)	WCS	*	*
									EDI	*	*

Note: BED = Binge Eating Disorder; BES = Binge Eating Scale [59]; BIAQ = Body Image Avoidance Questionnaire [60]; BITE = Bulimia Investigatory Test Edinburgh [61]; BN = Bulimia nervosa; BPS = Satisfaction and Dissatisfaction with Body Parts Scale [62]; BPSS = BSQ = Body Shape Questionnaire [63]; BULIT = Bulimia Test Revised [64]; Beck Depression Inventory [28]; CBT = Cognitive Behavioural Therapy; CES-D = The Center for Epidemiologic Studies Depression Scale [29]; DRES = Dutch Restrained Eating Scale [65]; EAT = Eating Attitudes Test [66]; EDDI = Eating Disorder Diagnostic Interview [53]; EDDS = Eating Disorder Diagnostic Scale [67]; EDE-Q = Eating Disorder Examination Questionnaire [68]; EDI = Eating Disorders Inventory [69]; EDNOS = Eating Disorder Not otherwise Specified; EWLB = Extreme Weight Loss Behaviors; IBSS = Ideal Body Stereotype Scale [70]; FFS = Forbidden Food Survey [71]; Hospital Anxiety and Depression Scale [32]; KDS = Kessler Distress Scale [31]; K-SADS Schedule for Affective Disorders and Schizophrenia for School-Age Children [30]; MADRS = Montgomery Asberg Depression Rating Scale [72]; OBE = Objective Binge Episodes; SATAQ = Sociocultural Attitudes Towards Appearance Questionnaire [73]; SIAB-EX = Structured Inventory for Anorexic and Bulimic Disorders [74]; TAU = treatment as usual; TFEQ = Three Factor Eating Questionnaire [75]; WCS = Weight and Shape Concerns Scale [76].

*p<.05 or lower, ns = non-significant, that is *p* > .05.

### Effect of interventions on eating disorder and depressive symptoms

Of the 27 studies, 24 (92%) revealed a statistically significant decrease in eating disorder symptoms while 12 (46%) revealed a statistically significant decrease in depressive symptoms. Eleven studies (42%) significantly decreased both depressive and eating disorder symptoms.

None of our hypothesized moderators of program success on depressive symptoms were supported. Programs with content targeting negative emotions or negative cognitions were not more likely to decrease depressive symptoms. Similarly, programs implementing CBT techniques were not more likely to decrease depressive symptoms. Furthermore, initial levels of depressive or eating disorder symptoms, age, and number of sessions also revealed no association with intervention efficacy in decreasing depressive symptoms (Table 2).

**Table 2:** **Characteristics of studies according to their success in decreasing depressive symptoms**

		**Decreases in depressive symptoms** ^**a**^		**Decreases in both depressive and eating disorder symptoms** ^**b**^	
	**Range**	**Significant decrease**	**No significant decrease**	**Significant decrease**	**No significant decrease**
		**(12 studies)**	**(15 studies)**	**(11 studies)**	**(16 studies)**
**Participant age [mean years, (SD)]**	14.4-46.5	27.2 (9.8)	28.7 (10.5)	25.4 (8.8)	33.3 (10.7)
**Number of sessions[mean (SD)]**	4-28	7.1 (2.7)	10.1 (7.1)	6.7 (2.4)	10.4 (7.1)
**Initial depressive symptoms percentile rank [mean rank(SD)]** ^**c**^	55-99	88.1 (6.9)	85.4 (13.4)	87.1 (7.8)	86.3 (13.1)
**Use of a screening cut-off score , N (%)**		7 (58%)	10 (67%)	6 (55%)	11 (69%)
**Face to face format, N (%)**		8 (67%)	10 (67%)	9 (82%)	9 (56%)
**CBT theoretical framework, N (%)**		9 (75%)	8 (53%)	8 (73%)	9 (56%)
**Content targeting negative emotions and cognitions, N (%)**		9 (75%)	10 (37%)	9 (82%)	10 (62%)

^a^For the 12 studies that found significant decreases in depressive symptoms, and the 15 that found no significant decrease, the mean age of participants, the mean depressive symptoms percentile rank at baseline, and the number of studies using a cut-off score, face-to face format, CBT framework, and content targeting emotions and cognitions are presented.

^b^The same information as in column ^a^is provided for the 11 studies that found significant decreases in both depressive and eating disorder symptoms, and the 16 that did not.

^c^Higher rank indicates that the participants in the study reported higher initial levels of depression.

Similarly, none of the hypothesized moderators were able to distinguish between programs that successfully decreased both depressive and eating disorder symptoms, and those that were successful in decreasing only one, or none (Table 2). However, there was a trend for number of sessions, t(24) = 1.75, p = 0.092, with interventions with fewer sessions tending to be more likely to conjointly decrease eating disorder and depressive symptoms.

## Discussion

The aim of the present study was to review the efficacy of indicated prevention and early interventions programs in decreasing concurrent depressive and eating disorder symptoms. Overall, the findings revealed that while the majority of interventions were successful in achieving decreases in eating disorder symptoms, fewer than half of the interventions identified were successful in decreasing both eating disorder and depressive symptoms. These results suggest that existing interventions may be limited in their overall impact and their long-term effects as remaining depressive symptoms could increase the likelihood of later recrudescence in eating disorder symptomatology [1]. Furthermore, these findings highlight the need for increased attention to comorbid depressive symptoms in intervention development and evaluation.

The findings from our review reveal that while 92% of the interventions resulted in significant decreases in eating disorder symptoms, only 42% were successfully in decreasing concurrent depressive and eating disorder symptoms. An important consideration lies in the fact that all of the interventions identified were primarily designed to target eating disorder symptoms, thus publication bias might have affected these ratios and made it more unlikely for our review to identify interventions successful only in decreasing depressive symptoms. Nevertheless, despite the seemingly widespread recognition of the important role of depressive symptoms in eating disorder pathology, illustrated by the frequent inclusion of depressive symptoms as a secondary outcome and the consideration of the effects of eating disorder interventions on depressive symptoms or negative affect in meta-analyses of eating disorder prevention interventions [19], our findings suggest that eating disorder interventions reveal a limited capacity to decrease depressive symptoms. Interestingly, while their importance as a secondary outcome is recognized, the failure to impact depressive symptoms seems to have received little attention. One potential explanation could be a lack of clarity in the conceptualization of depressive symptoms within intervention evaluation. Depressive symptomatology might be assessed as a control factor that could moderate intervention effects, and consequently the lack of intervention effects on this dimension would not be interpreted as a limitation of the intervention success. However, the studies included in the present review assessed for change in depressive symptoms, and frequently referred to it as a secondary outcome, suggesting that the intervention had been hypothesized to positively impact depressive symptoms.

The limited success of the interventions included in this review in decreasing depressive symptoms given their general success in decreasing eating disorder symptoms, suggests that, consistent with theories of comorbidity in a subset of individuals at least, depressive symptoms may not be secondary to eating disorder symptomatology [10]. Some authors have suggested that individuals suffering from eating disorder symptoms may experience depressive symptoms due to the burden of the illness [5]. However, the finding that the improvement of eating disorder symptomatology was not robustly associated with improvement in depressive symptoms, suggests this may not be the only mechanism accounting for the comorbidity and supports theories highlighting common etiological factors [10].

Overall, our proposed moderating factors were not predictive of success in decreasing depressive symptoms. One potential explanation for this failure is that we lacked statistical power due to using dichotomous variables as outcomes and for many moderators, or that the continuous variables assessed lacked variability. However, it might also be that variables other than those assessed in these studies, predict intervention effects on depressive symptoms. Our finding did reveal a trend-level finding regarding number of sessions, with shorter interventions revealing a higher likelihood of decreasing both eating disorder and depressive symptoms. This finding is consistent with those of meta-analytic reviews of interventions targeting depressive symptoms among children and adolescents [77]. These authors hypothesized that engagement may be higher with shorter programs, accounting for the stronger effects.

Our study was limited by some aspects of the available data. We used participant recruitment criteria (self-selected versus screened) as a proxy for eating disorder severity in part due to the wide variability in eating disorder instruments used and the lack of available normative data for some of those instruments. Using a more sensitive measure of eating disorder severity might have produced different results. Furthermore, while some studies provided rates of diagnosable depression in their sample of baseline, rates of depression at post-test were not available, precluding examination of decreases in diagnosable depression.

Nevertheless, these findings reveal the gap in knowledge and practice regarding the treatment of concurrent eating disorder and depressive symptoms and suggest some directions for future work:

1. Increasing our understanding of the course of depressive symptoms in relation to eating disorder symptoms during interventions might help clarify the mechanism of change in depressive symptoms and identify individuals benefiting less from the intervention effects on depressive symptoms.
2. Exploring participants’ experience of the causes of depressive symptoms, in particular as related or unrelated to their eating disorder symptoms, would contribute to increasing our understanding of the partial success of eating disorder interventions in decreasing concurrent eating disorder and depressive symptoms.
3. The development and evaluation of depression-specific modules in eating disorder interventions would provide evidence of increased efficacy in decreasing depressive symptoms.
4. Including measures of eating disorder symptoms in interventions primarily targeting depressive symptoms could further inform interventions for these comorbid concerns, and contribute to greater treatment efficiency.

## Conclusions

Eating disorder and depressive symptoms are frequently comorbid and, in recognition of this, eating disorder interventions have often assessed depressive symptoms. The effect indicated prevention and early intervention programs on depressive symptoms is somewhat limited, however, and the characteristics of successful interventions are unclear. Further efforts are required to develop interventions that are successful in decreasing both eating disorder and depressive symptoms in order to increase treatment efficiency and the maintenance of therapeutic effects.
